# Quarantine at home may not enough!-from the epidemiological data in Shaanxi Province of China

**DOI:** 10.1186/s13104-020-05342-5

**Published:** 2020-11-04

**Authors:** Lei Shi, Qian Li, Kang Li, Jie Zheng, Yingli He, Xi Zhang, Xianfeng Gong, Wei Wang, Qing Zhang, Chao Dai, Wenxuan Zhao, Xuefei Meng, Feng Du, Pei Fan, Chunyan Li, Chunyan Gao, Yuan Yang, Xiaojing Liu, Yunru Chen, Jinfeng Liu, Jianzhou Li, Nan Yang, Yinghua Niu, Hongmei Chen, Guoyu Zhang, Taotao Yan, Li Zhu, Qunying Han, Wanhu Fan, Feng Ye, Zhengwen Liu, Shumei Lin, Yingren Zhao, Tianyan Chen

**Affiliations:** 1 Division of Infectious Disease, The First Affiliated Hospital of Xi’an Jiaotong University, No. 277 Yanta West Road, Xi’an, 710061 Shaanxi China; 2 Xi’an CDC, Shaanxi, 710000 China; 3 Xi’an Chest Hospital, Shaanxi, 710100 China; 4 The Central Hospital of Ankang, Shaanxi, 725000 China; 5 The Central Hospital of Hanzhong, Shaanxi, 723000 China; 6 The Second People’s Hospital of Hanzhong, Shaanxi, 723000 China; 7 The Central Hospital of Weinan, Shaanxi, 714000 China; 8 The Central Hospital of Baoji, Shaanxi, 721008 China; 9 The People’s Hospital of Tongchuan, Shaanxi, 727000 China; 10 The Second People’s Hospital of Yanan, Shaanxi, 716004 China; 11 The Central Hospital of Shangluo, Shaanxi, 726000 China

**Keywords:** SARS-CoV2, COVID-19, Epidemiological features, Family cluster

## Abstract

**Objectives:**

A pneumonia associated with 2019 novel coronavirus (2019-nCoV, subsequently named SARS-CoV2) emerged worldwide since December, 2019. We aimed to describe the epidemiological characteristics of 2019 coronavirus disease (COVID-19) in Shaanxi province of China.

**Results:**

1. Among the 245 patients, 132 (53.9%) were males and 113 (46.1%) were females. The average age was 46.15 ± 16.43 years, ranging from 3 to 89 years. 2. For the clinical type, 1.63% (4/245) patients were mild type, 84.90% (208/245) were moderate type, 7.76% (19/245) were severe type, 5.31% (13/245) were critical type and only 0.41% (1/245) was asymptomatic. 3. Of the 245 patients, 116 (47.35%) were input case, 114 (46.53%) were non-input case, and 15 (6.12%) were unknown exposure. 4. 48.57% (119/245) cases were family cluster, involving 42 families. The most common pattern of COVID-19 family cluster was between husband and wife or between parents and children.

## Introduction

Since late December 2019, a novel coronavirus disease, later named COVID-19 by World Health Organization (WHO), has emerged in Wuhan, China [1–3], and then spread to other provinces in China and the world. As of July 17, 2020, nearly 13,810,534 confirmed cases and 590,005 death cases have been reported in almost all countries and territories.

Wuhan city has been closed since January 23, 2020, in the following 2 days, the Chinese government mobilized all forces to stop the epidemic, including the suspension of public transport, subway, ferry and long-distance passenger transport, and the temporary closure of airports and trains. Shaanxi is located in the northwest of China, bordering Wuhan in the southeast, covering an area of 205,800 square kilometers, with 10 cities and a permanent population of 38.64 million. It is very important to understand the epidemiological characteristics of COVID-19 transmission outside Wuhan for the effective control strategies.

During major infectious disease outbreaks, quarantine always be the necessary preventive measure, but experience about COVID-19 pandemic is limited. During the past several months, to slow down the spread of coronavirus, countries around the world have been implementing various measures, including “social distancing,” “work at home,” “quarantine at home,” “centralized isolation,” “lockdown” and so on. Most countries in COVID-19 take the measure of quarantine at home for diagnosed individuals, patients with severe symptom were then sent to hospital. Some countries take the measure of centralized isolation. In China, centralized isolation is adopted for all SARS-CoV-2 nucleic acid positive individuals. Quarantine at home can reduce the risk of community communication but increases the risk of transmission within the family [4]. Centralized isolation can reduce COVID-19 mortality and reduced the rate of growth in hospitalizations [5].

In this study, we described the epidemiological characteristics of COVID-19 cases in Shaanxi province of China. Totally, 245 cases were reported in 10 cities of Shaanxi from January 23, 2019 to February 22, 2020 in China. We hope to provide valuable information for the public health policy makers from COVID-19 outbreak area.

## Main text

### Methods

#### Patients

This is a retrospective study, all the patients admitted in Shaanxi province. Totally, 245 COVID-19 patients were enrolled in this study from January 23, 2020 to February 22, 2020. The diagnosis was confirmed by RT-PCR for nasopharyngeal swab nucleic acid.

#### Data source

Since January 23, 2020, the Centers for Disease Control and Prevention (CDC) of Shaanxi Province has started the COVID-19 investigation. De-identified data were extracted from the case report system of Health Commission of Shaanxi Province. The personally identifiable information of all subjects were removed to protect personal privacy. The clinical type were also double checked with the doctors from hospitals Which treat the COVID-19 patients in Shaanxi Province.

#### Variable information

The demographic characteristics, onset time, diagnosis time and hospitalization treatment institutions were collected. If the patient returned in 14 days before the onset of the disease from a place where there has COVID-19 outside Shaanxi, they were classified as input exposure. All COVID-19 patients are classified as mild, moderate, severe and critically cases at admission, according to COVID-19 guidelines (the sixth version) made by National Health Commission of the People’s Republic of China. The clinical type was as follows:

Mild: Slight clinical symptoms, no pneumonia in imaging.

Moderate: Fever, respiratory tract and other symptoms, pneumonia in imaging.

Severe: Presenting any one of the followings:

1. Respiratory distress, respiratory rates ≥ 30 per minute;
2. Pulse oxygen saturation ≤ 93% at rest;
3. Oxygenation Index (PaO2/FiO2) ≤ 300 mmHg.

Pulmonary imaging progressed more than 50% in 24–48 h, regard as severe case.

Critical: Presenting any one of the followings

1. Respiratory failure with invasive ventilation;
2. Signs of shock (circulatory failure).
3. Failure of any other organ failure with ICU care.

For the epidemiological curve, the date of onset is defined as the date when the case reports fever or cough in the epidemiological investigation. The date of diagnosis refers to the date when the coronavirus nucleic acid test was positive. All cases are confirmed by virus nucleic acid test results. Asymptomatic patients indicate the novel coronavirus nucleic acid test positive, but no fever, cough and pulmonary imaging changes. The onset date of asymptomatic patients was defined as the positive date of virus nucleic acid test in laboratory.

#### Statistical analysis

The continuous measurements of normal distribution were represented as means (standard deviations, SDs), the non-normal distributions were showed as median values (Interquartile ranges, IQR), and the categorical variables were represented as subject number (percentage). We used GraphPad Prism 8 and ArcGIS Online for data analysis.

### Results

#### Gender and age distribution

**Fig. 1:**
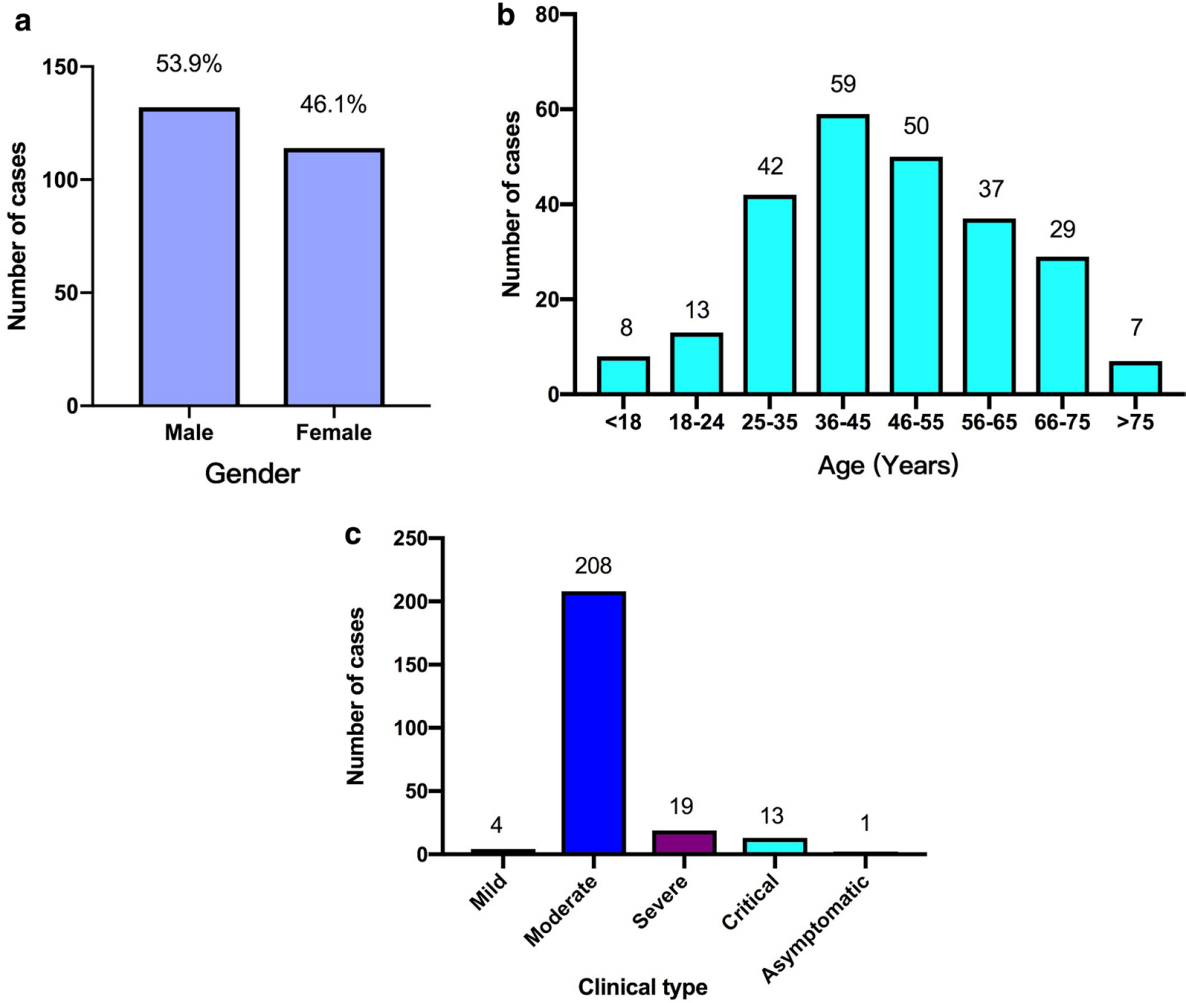
Gender and age distribution of COVID-19 patients in Shaanxi, China. **a** Gender distribution. **b** Age distribution. **c** Clinical type

The gender and age distribution of confirmed cases in Shaanxi province is shown in Fig. 1. Of the 245 patients 132 (53.9%) were males and 113 (46.1%) were females (Fig. 1a). The average age was between 46.15 ± 16.43 years, ranging from 3 to 89 years, the majority age is between 25 and 75 years old. Under 18, 18–24, 25–35, 36–45, 46–55, 56–65, 66–75 and over 75 years old accounted for 3.27% (8/245), 5.31% (13/245), 17.14% (42/245), 24.08% (59/245), 20.41% (50/245), 15.10% (37/245), 11.84% (29/245) and 2.86% (7/245) respectively (Fig. 1b). For the clinical type, mild type, moderate type, severe type and critical type accounted for 1.63% (4/245), 84.90% (208/245), 7.76% (19/245) and 5.31% (13/245). Only 0.41% (1/245) was asymptomatic (Fig. 1c).

#### Time distribution

**Fig. 2:**
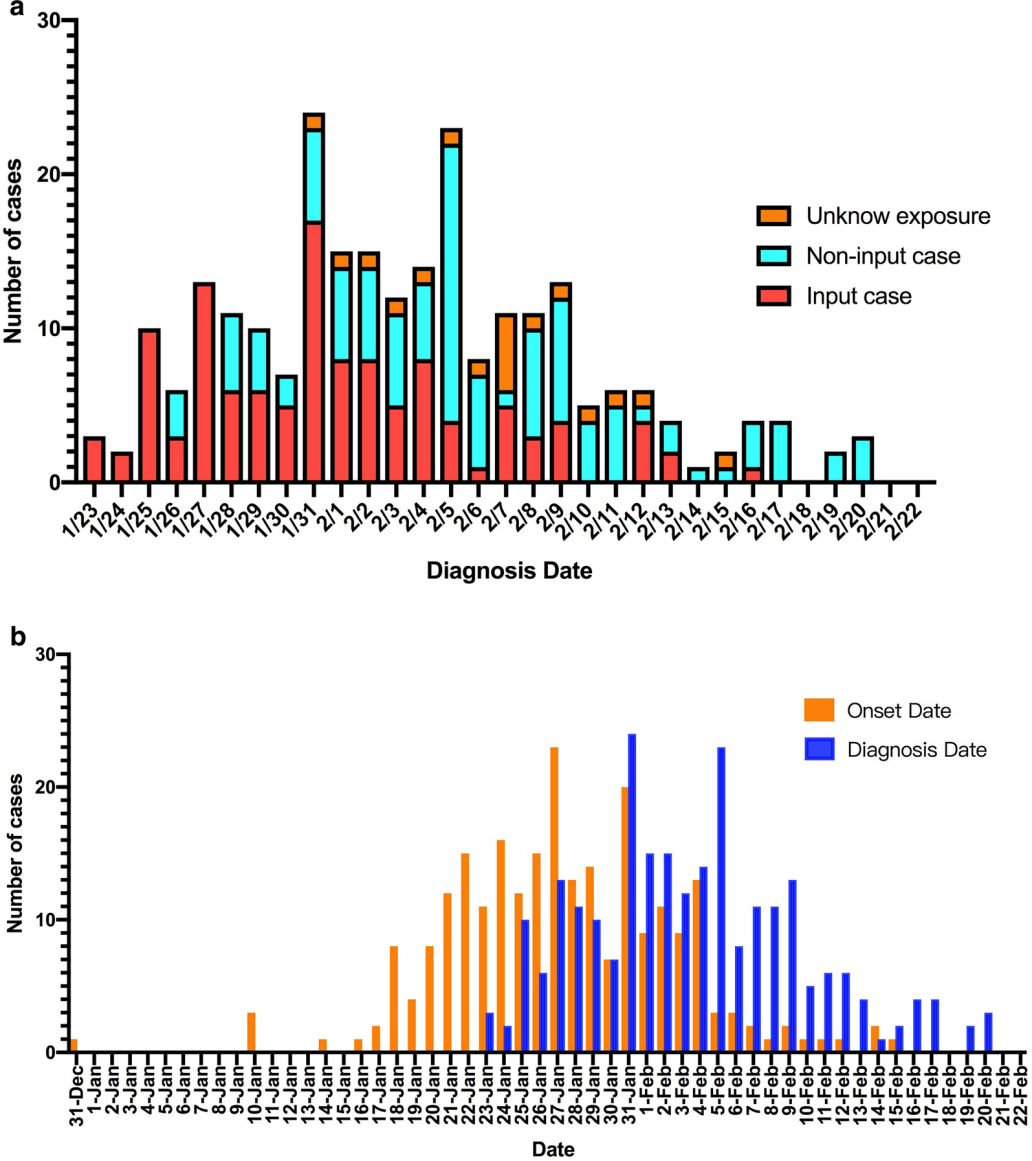
Time distribution of COVID-19 in Shaanxi, China. **a** Input case, non-input case and unknown exposure by diagnosis date. **b** Number of cases by onset and diagnosis date

Of the 245 patients, 116 (47.35%) were input case, 114 (46.53%) were non-input case, and 15 (6.12%) were unknown exposure. The data showed that in the early stage of epidemic, the input cases were the main type, and the input and local cases coexisted from the 4th day, lasting for 2 weeks. During this period, the input cases decreased and the local cases increased gradually, the local cases were the main type from the 3rd week. From the 27th day (February 18), there was no new case for the first time (Fig. 2a).

According to the epidemic curve drawn by the onset date, the number of patients increased gradually from January 14, peaked on January 27, and then decreased gradually. The epidemic curve by the diagnosis date shows that the number of reported cases reached the first peak on January 27, and had two single day high values on January 31 and February 5, then decreased slowly. There was no new cases for the first time on February 18 (Fig. 2b).

#### Geographical distribution by time

**Fig. 3:**
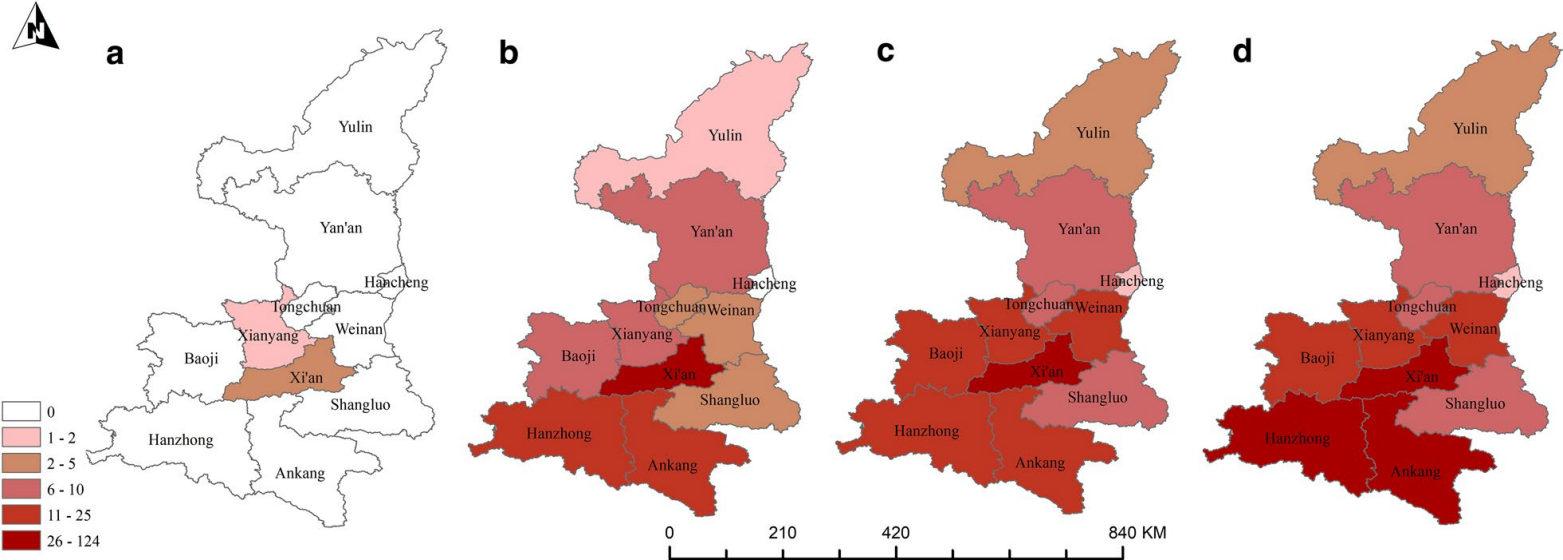
Geographical distribution by time of COVID-19 patients in Shaanxi, China. **a** By January 23, 2020. **b** By February 1, 2020. **c** By February 11, 2020. **d** By February 22, 2020. Drawn by ArcGIS Online, https://www.esri.com/zh-cn/arcgis/products/arcgis-online/

Based on the retrospective analysis of the date and geographical location of the cases, the geographical distribution of coronavirus pneumonia in four different periods after the closure of Wuhan was observed in Shaanxi Province. Of all the cases, 1.22% (3/245) cases were before January 23, 2020 (Fig. 3a), accounting for 2/12 cities in Shaanxi. 41.22% (101/245) of cases occurred before February 1, 2020, accounting for 11/12 cities in Shaanxi (Fig. 3b). 89.39% (219/245) of cases occurred before February 11, 2020 (Fig. 3c) and all the cases occurred before 22 February 2020, accounting for all the cities in Shaanxi (12/12) (Fig. 3d). It shows that 10 days after the closure of Wuhan, the confirmed cases have been distributed in most cities in Shaanxi Province.

#### Cluster and Family cluster analysis

The cluster refers to COVID-19 cases with a clear history of exposure in epidemiological history. The family cluster is defined as epidemiology found that there were ≥ 2 patients of COVID-19 in a family related in time and place. Of the 245 patients, there were 60.28% (149/245) cluster cases (Additional file 1: Fig. S1A). The frequency of cluster occurs at 2 persons, 3 persons, 4 persons, 5 persons, more than 5 people were 20 times (40 people), 13 times (39 people), 7 times (28), 3 times (15 people) and 3 (27 people) (Additional file 1: Fig. S1B, C). There were totally 42 family cluster events, involving 119 people, accounting for 48.57% (119/245) of the total number (Additional file 1: Fig. S1D). The most common pattern of COVID-19 family cluster was between husband and wife or between parents and children (Additional file 1: Fig. S1E, F). Some typical examples of family cluster are showed in Additional file 2: Fig. S2.

### Discussion

This is a descriptive study on epidemiology characteristics of the COVID-19. It presents the novel coronavirus pneumonia incidence in Shaanxi province within 1 month after the closure of Wuhan. Totally, 245 cases were reported in Shaanxi, China. It needs to be explained that these situations have taken place under the strict prevention and control measures, indicating the control measures were effective in Shaanxi.

2019 novel coronavirus is the seventh known coronavirus that can infect humans. The other six coronavirus are HCoV-229E, HCoV-OC43, HCoV-NL63, HCoV-HKU1 and SARS-CoV [6], which cause severe acute respiratory syndrome [7], and MERS-CoV, leading to Middle East respiratory syndrome [8]. SARS-CoV was first appeared in Guangdong, then spread to 24 provinces in China and 28 other countries in the world [9–11]. MERs-Cov was first identified in Saudi Arabia in 2012. Since 2012, MERS has spread to 27 countries in the world, including the Middle East, Asia and Europe [12]. We found that, like SARS and MERS, all the population were susceptible to 2019 novel coronavirus, but most of the patients showed moderate symptoms in COVID-19. This is consistent with the conclusions of other researchers [13, 14].

It is worth noting that of the 24 patients who were classified as mild type on the diagnosis date, 83.33% (20/24) have fever, cough and pulmonary imaging changes in the following hospitalization days. 17 cases among them were corrected to moderate type, 2 cases to severe type, and 1 case to critical type during the hospitalization. Of the 245 patients, only 0.41% (1/245) was asymptomatic. It is suggested that the symptoms or imaging examination on diagnosis day can’t reflect the severity of the disease.

We retrospectively analyzed the novel coronavirus pneumonia incidence in Shaanxi province. January 23, 2020 was the first day to detect coronavirus nucleic acid in Shaanxi Province, and December 31, 2019 was the first day of symptoms in confirmed patient. Considering the first four patients with symptoms have returned after the onset in Wuhan (returned on 21 January, 14 January, 15 January, 19 January), and the first patient with symptoms in Shaanxi returned on January 7, he has onset on January 14 and has diagnosed on January 25. It is estimated that the first patient in Shaanxi Province appeared on January 7, 2020. It is worth noting that the peak time for symptoms was 19 days later.

Of the 245 patients, there are 42 family cluster events, accounting for 48.57% of the total number, indicating COVID-19 is easily transmitted among family members. We found that the most common pattern of COVID-19 family cluster was between husband and wife or between parents and children, showing close contact is the most important mode of transmission. This is consistent with other articles [15, 16]. Considering that there are many ways of transmission of the coronavirus, and viral nucleic acids have already been found in blood, urine, feces and conjunctival secretions and the respiratory tract [17, 18]. If suspected or mild patients quarantined at home., it may increase the risk of transmission in family. The government should consider centralized isolation when make the prevention and control measures, so as to minimize the risk of disease transmission.

### Conclusion

In conclusion, we described and analyzed the epidemiological characteristics of 245 COVID-19 cases in Shaanxi province of China. The COVID-19 infection was of family clustering onset in Shaanxi, the suspected or mild patients may have the risk of spreading disease if they were quarantined at home. Although the majority of patients were moderate cases, strict centralized isolation had proven to be the most effective measure to control new cases. We hope to provide valuable reference information for the public health policy makers from novel coronavirus pneumonia outbreak area.

## Limitation

There are also several limitations in this study. First of all, the study was conducted in an area outside of Wuhan, with some regional restrictions, where the total number of cases was relatively small. Secondly, in the epidemiological investigation of the cases, there may be the problem of memory bias for symptoms and contact history.

## Supplementary information

Supplementary file 1 (JPG 5764 kb)

Supplementary file 2 (JPG 6134 kb)

## Data Availability

The datasets analyzed in the current study will be available from the corresponding author upon reasonable request.

## Abbreviations

2019-nCoV: 2019 novel coronavirus
COVID-19: 2019 coronavirus disease
WHO: World Health Organization
CDC: Centers for Disease Control and Prevention
SDs: Standard deviations
IQR: Interquartile ranges
