# Supplementary figures and images for: Quarantine at home may not enough!-from the epidemiological data in Shaanxi Province of China
Source: BMC Res Notes. 2020 Nov 4;13:506. doi: 10.1186/s13104-020-05342-5 (PMC7661832; doi:10.1186/s13104-020-05342-5)

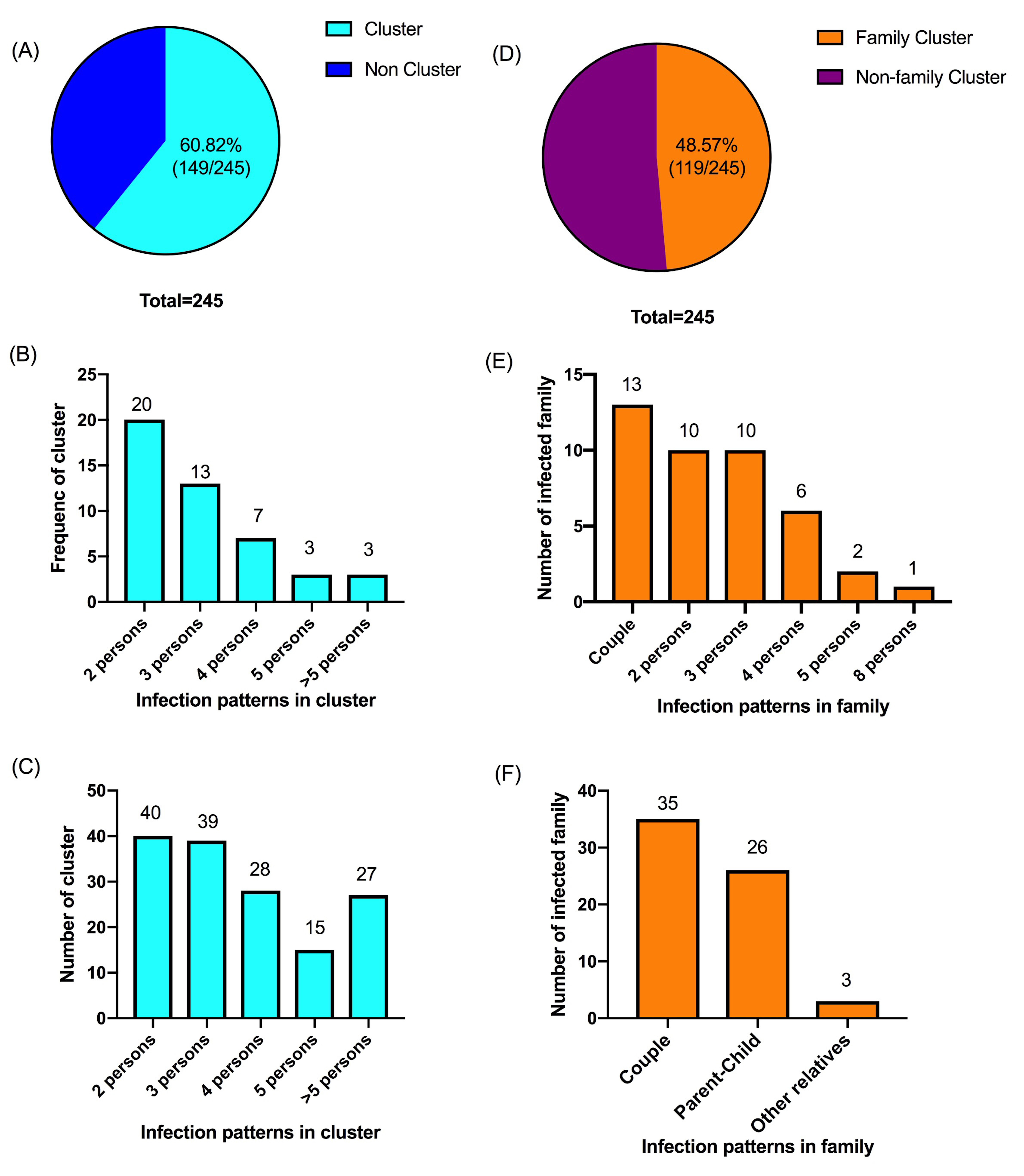

Supplement: Supplementary file 1 — (JPG 5764 kb) [file 13104_2020_5342_MOESM1_ESM.jpg]
